# Product development and quality of pharmacy compounded chenodeoxycholic acid capsules for Dutch cerebrotendinous xanthomatosis patients

**DOI:** 10.3389/fphar.2023.1264997

**Published:** 2023-10-17

**Authors:** Natalja Bouwhuis, Bart A. W. Jacobs, E. Marleen Kemper

**Affiliations:** ^1^ Department of Pharmacy and Clinical Pharmacology, Amsterdam UMC, Amsterdam, Netherlands; ^2^ Platform Medicine for Society, Amsterdam UMC, Amsterdam, Netherlands; ^3^ Department of Experimental Vascular Medicine, Amsterdam UMC, Amsterdam, Netherlands

**Keywords:** pharmacy preparation, pharmacy compounding, chenodeoxycholic acid, cerebrotendinous xanthomatosis, product validation, stability, good manufacturing practice, European Pharmacopoeia

## Abstract

**Introduction:** In 2017 the drug chenodeoxycholic acid (CDCA) became unavailable to Dutch patients with the rare inborn error of metabolism cerebrotendinous xanthomatosis (CTX). This was a direct result of a steep price increase after CDCA was authorized in the EU as an orphan drug. As a result, Dutch health insurance companies were unable to reimburse this drug and the availability of CDCA to patients with CTX was directly at risk creating an unmet medical need. CTX is characterized by juvenile cataract, tendon xanthomas, infantile-onset diarrhea, psychomotor retardation and progressive cerebellar ataxia. Treatment with CDCA, when initiated before neurological symptoms are present, can prevent the onset of neurological complications.

**Methods:** To assure continuation of patient treatment with a high quality product, the hospital pharmacy of the Amsterdam UMC developed CDCA capsules as a pharmacy preparation. A simple and robust formulation was developed for capsules in a broad dose range of 35–250 mg, ensuring that both pediatric and adult patients can receive an exact dose tailored to their specific needs. Capsules are prepared manually on a small scale for the individual patient. To assure the quality of the product, product validation and stability studies were performed.

**Results:** The results show that the product complies with all specifications based on the requirements of the European Pharmacopoeia. The capsules contain the declared amount of CDCA, no degradation product or other (microbiological) impurities are formed during the production process and the capsules show a quick dissolution profile. Stability studies indicate that it is a stable product and no impurities increase or arise over time. These results show that these pharmacy preparations are of high quality and comply to Good Manufacturing Practice (GMP) requirements.

**Discussion:** Through our research, we have demonstrated that pharmacy compounding can be a viable alternative in situations where immediate access to essential medication is crucial or when certain drugs are temporarily inaccessible. The purpose of this paper is to offer comprehensive guidance to other pharmacies to improve the availability of currently inaccessible drugs through the practice of pharmacy compounding, thereby facilitating improved patient care.

## 1 Introduction

Chenodeoxycholic acid (CDCA) is an effective drug in the treatment of cerebrotendinous xanthomatosis (CTX) (Federico and Gallus, 2003). CTX is a rare metabolic disease caused by *CYP27A1* gene mutations leading to reduced plasma levels of bile acids including CDCA, and accumulation of toxic bile acid intermediates such as cholestanol in plasma and tissues. Due to these toxic substances, patients may experience various, often severe, symptoms such as infantile-onset diarrhea, juvenile cataract and tendon xanthomas, and adult onset of neurologic dysfunction (including psychiatric disturbances, cerebellar symptoms, neuropathy and dementia) (Federico and Gallus, 2003). Treatment with CDCA, when initiated before neurological symptoms are present, can prevent the onset of neurological complications (Stelten et al., 2022).

Initially, orally administered CDCA was used to dissolve gallstones. However, this indication has become obsolete (Fiorucci and Distrutti, 2019). Since the 1970s, CDCA treatment is used in CTX patients in the Netherlands in an off-label setting (Dutch National Health Care Institute, 2018). In 2017, CDCA was approved by the European Medicines Agency as an orphan drug for the treatment of CTX (Leadiant GmbH, 2017). After market authorization, the price increased from €30.000 to a list price of around €170.000 per patient per year (Sheldon, 2018; KNMP, 2023). As a consequence of this price increase, the secretary of Healthcare did not provide for a legal basis for reimbursement and Dutch health insurance companies were unable to reimburse the drug, other than a payment by way of courtesy. As a result an essential treatment was no longer available to CTX patients in the Netherlands (Sheldon, 2018).

In order to prevent treatment interruption and to assure CDCA availability, the hospital pharmacy of the Amsterdam UMC developed CDCA capsules by pharmacy preparation, also known as pharmacy compounding or *formula magistralis* (Sheldon, 2018). Pharmacy compounding is often applied when authorized medicines are not available or not suited for patient treatment, for example, when a specific dose is required. The authorized CDCA product, which was not reimbursed, is only available as 250 mg capsules. This formulation is suitable for adult dosing, as they receive a starting dose of 750 mg per day in three divided doses, which can be increased to 1,000 mg per day. However, pediatric patients receive a starting dose of 5 mg/kg per day, in three divided doses, which can be increased to 15 mg/kg per day (Leadiant GmbH, 2017). The smallest required dose for Amsterdam UMC patients was 105 mg per day in three divided doses of 35 mg. CDCA 250 mg capsules are therefore unsuitable for accurate dosing in pediatric patients. On top of that, the large capsule size can be difficult to swallow for pediatric patients. In order to make CDCA capsules available for both pediatric and adult patients, the CDCA capsules were developed and manufactured in a range of 35–250 mg, following applicable good manufacturing practice (GMP) as well as national compounding guidelines (KNMP, 2022). Many hurdles were tackled during this development which are discussed extensively in an article by Polak et al. (2021). In this article we will elaborate on the product validation and stability studies that followed, to demonstrate the quality of the pharmacy compounded CDCA capsules.

## 2 Materials and methods

### 2.1 Starting materials

The product formulation consists of the active pharmaceutical ingredient (API) CDCA, the excipient lactose monohydrate (when needed), the lubricant silica (colloidal anhydrous) and clear hard gelatin capsules. All used starting materials complied to the specifications of the European Pharmacopoeia (Ph.Eur.). CDCA is a white or almost white powder, with molecular formula C_24_H_40_O_4_ and a molecular weight of 392.6 g/mol. The powder is very slightly soluble in water and freely soluble in ethanol (96 per cent), as described in the Ph.Eur. Ph.Eur. reference standards are used in Quality Control. The capsules were packed in pharmaceutical grade HDPE DUMA Twist-Off containers with PP screw caps. All materials were procured from qualified suppliers.

### 2.2 Pharmacy compounding

Due to bad flowing properties of the CDCA API, 0.5% (w/w) silica was added to the formulation. Depending on CDCA dosage and capsule size, lactose monohydrate was used as a filler substance where needed. The capsules were prepared making a dry powder blend with mortar and pestle, mixing the compounds in equal parts until a homogeneous mixture was created. The powder mixture was manually distributed over the needed amount of capsules using dedicated capsule filling machines, in which a maximum of 100 capsules can be filled simultaneously. Therefore all batch sizes are a multiple of 100 capsules. The following in-process control steps (test that are performed during manufacturing) were incorporated throughout the process: calculation of the weight distribution (RSD) and the deviation from the theoretical weight. This manual production process of pharmacy compounding of capsules is a validated process in the Amsterdam UMC pharmacy, assuring that the capsules are reproducible and consistent.

### 2.3 Validation batches

Product validation was performed on predetermined worst-case product doses. Selection factors included were the smallest and largest capsule size, the smallest and largest ratio of lactose monohydrate vs. CDCA API, and the lowest and the highest dose. Based on this, 35 mg CDCA in size 3 capsules and 250 mg CDCA in size 0 capsules were selected as the worst-case products. All doses are summarized in Table 1. The highest dose of 250 mg CDCA with 0.5% silica completely fills the largest capsule size (size 0) and therefore requires no additional lactose. For the lowest dose, 35 mg CDCA API, to fit in the smallest available size 3 capsule, addition of around 80% filler substance lactose monohydrate was needed, which was the highest percentage of lactose of all doses that were being made. For both doses, three validation batches were manufactured, according to European GMP guidelines and national compounding guidelines. During manufacturing, all portions of 100 capsules of a batch were mixed before filling in the containers to assure a homogeneous batch.

**TABLE 1:** Composition of all doses of compounded CDCA capsules and worst-case selection for product validation.

CDCA API (dose)	Silica	Lactose monohydrate	Capsule size	Worst case	Batch size for validation
35 mg	0.5%	Approximately 80%	3	Yes	1,300 capsules
40 mg	0.5%	Approximately 80%	3	No	-
45 mg	0.5%	Approximately 75%	3	No	-
50 mg	0.5%	Approximately 70%	3	No	-
75 mg	0.5%	Approximately 55%	3	No	-
80 mg	0.5%	Approximately 50%	2	No	-
90 mg	0.5%	Approximately 70%	2	No	-
100 mg	0.5%	Approximately 50%	2	No	-
120 mg	0.5%	Approximately 35%	2	No	-
140 mg	0.5%	Absent	2	No	-
200 mg	0.5%	Approximately 30%	0	No	-
250 mg	0.5%	Absent	0	Yes	1,000 capsules

### 2.4 Quality Control

Product specifications were based on the requirements in Ph.Eur. monographs 2619 *Pharmaceutical Preparations* and 0016 *Capsules* and ICH guideline Q6A *Specifications: Test Procedures and Acceptance Criteria for New Drug Substances and New Drug Products: Chemical Substances* and can be found in Table 2 (ICH, 1999). The impurities and acceptance limits were selected based on individual Ph.Eur. monograph 1189 *Chenodeoxycholic Acid*. No additional impurities were expected due to the production process. The high pressure liquid chromatography with differential refractometer (HPLC-RI) analytical method from individual Ph.Eur. monograph 1189 *Chenodeoxycholic Acid* was used.

**TABLE 2:** Product specifications of CDCA capsules according to Ph.Eur.

Test	Specification	Method	References
Appearance	Clear capsule with white or almost white powder	Visual	ICH Q6A, Ph.Eur. 2619
Identity (HPLC)	Positive	Ph. Eur. 2.2.29	ICH Q6A, Ph.Eur. 2619
Related substances (HPLC)		Ph. Eur. 2.2.29	ICH Q6A, Ph.Eur. 2619
-Impurity A (ursodeoxycholic acid)	NMT 1%		
-Impurity B (cholic acid)	NMT 0.5%		
-Impurity C (lithocholic acid)	NMT 0.1%		
-Impurity H (3α,7β-dihydroxy-12-oxo-5β-cholan-24-oic acid)	NMT 0.2%		
-Impurity I (3α-((3α,7α-dihydroxy-24-oxo-5β-cholan-24-yl)oxy)-7α-hydroxy-5β-cholan-24-oic acid)	NMT 0.5%		
-Any other impurity	NMT 0.25%		
-Total impurities	NMT 1.5%		
Assay (HPLC)	90.0%—110.0%	Ph. Eur. 2.2.29	ICH Q6A, Ph.Eur. 2619
Uniformity of dosage units	AV ≤ 15	Ph. Eur. 2.9.40	ICH Q6A, Ph.Eur. 2619, Ph.Eur. 0016
Microbiology			ICH Q6A, Ph.Eur. 2619
-TAMC	NMT 10^3^ CFU/g	Ph. Eur. 2.6.12	
-TYMC	NMT 10^2^ CFU/g	Ph. Eur. 2.6.12	
-E. Coli	Absent	Ph. Eur. 2.6.13	
Dissolution	≥ 80% at 30 min	Ph. Eur. 2.9.3; medium phosphate buffer pH6.8, assay by HPLC (see assay)	ICH Q6A, Ph.Eur. 0016
-at 5, 10, 15, 20, 30 min			
Disintegration	< 30 min	Ph. Eur. 2.9.1; medium water, with disc	ICH Q6A, Ph.Eur. 0016

Additional analytical method validation on specificity and accuracy was performed for identification and quantification of CDCA and its related substances in CDCA capsules. The acceptance criteria for specificity included: all peaks in the chromatograms should be assigned (retention times); in the injection of standard solutions and sample, the active compound CDCA and the specified impurity should be separated with a resolution ≥15%; in the mobile phase no significant peak above reporting threshold should be present which might interfere with CDCA and the known impurity; the retention time of the sample solution CDCA should be between 95%—105% of the retention time of the standard solution. The acceptance criteria for accuracy was a mean recovery of 98%–102% with RSD ≤2%.

### 2.5 Stability program

The stability program was performed on the validation batches, consisting of three batches of both doses, as required per ICH guideline Q1A (R2) *Stability testing of new drug substances and drug products* (ICH, 2003). The long term stability study at 25°C ± 2°C and 60%RH ±5%RH included testing at time points: 0, 3, 6, 9 and 12 months. The accelerated stability study at 40°C ± 2°C and 75%RH ± 5%RH included testing at time points: 0, 3 and 6 months. At time points 3 and 9 months, reduced testing was performed as not all tests are stability indicating parameters and these were not final time points on which the shelf life would be determined. The tests that were performed at each time point are shown in Table 3.

**TABLE 3:** Stability program of CDCA capsules (three 35 mg batches and three 250 mg batches). Long-term conditions are set at 25°C ± 2°C and 60%RH ± 5%RH and accelerated conditions at 40°C ± 2°C and 75%RH ± 5%RH.

Test	T = 0	Accelerated		Long-term			
		T = 3	T = 6	T = 3	T = 6	T = 9	T = 12
Appearance	X	X	X	X	X	X	X
Identity	X	X	X	X	X	X	X
Related substances	X	X	X	X	X	X	X
Assay	X	X	X	X	X	X	X
Uniformity of dosage units	X	-	X	-	X	-	X
Microbiology	X	X	X	X	X	X	X
Dissolution	X	-	X	-	X	-	X
Disintegration	X	-	X	-	X	-	X

## 3 Results

### 3.1 Product validation

Quality Control results of the product validation show that for both 35 and 250 mg CDCA capsules all six batches complied with the set specifications and therefore complied with Ph.Eur. requirements. All results are shown in Table 4. The products contained the claimed amount of CDCA, with a range of 100.1%–105.5% (specification: 90%–110%). There was little inter-batch variation. The mean assay content of the 35 mg and the 250 mg capsules were 100.8% and 104.7%, respectively. The capsules showed a rapid and reproducible dissolution profile; all batches showed more than 95% dissolution within 15 min and showed little inter-batch variation and variation between the two doses, as shown in Table 4. The test for uniformity of dosage units complied with an acceptance value (AV) ranging from 6 to 13.2 (specification: ≤15), which confirmed that the CDCA API was distributed evenly over the capsules. The capsules had a quick disintegration of ≤3 min (specification: ≤30 min). No degradation products or (microbiological) impurities had formed during the production process. Based on these results, the production process of manufacturing CDCA capsules in a range of 35–250 mg was considered validated.

**TABLE 4:** Results product validation CDCA capsules 35 mg and 250 mg.

		35 mg capsules			250 mg capsules		
Test	Specification	Batch 1	Batch 2	Batch 3	Batch 1	Batch 2	Batch 3
Appearance	Clear capsule with white to broken white powder	Complies	Complies	Complies	Complies	Complies	Complies
Identity (HPLC)	Positive	Positive	Positive	Positive	Positive	Positive	Positive
Related substances (HPLC)							
-Impurity A	NMT 1%	0.00%	0.00%	0.00%	0.00%	0.00%	0.00%
-Impurity B	NMT 0.5%	<0.05%	<0.05%	<0.05%	<0.05%	<0.05%	<0.05%
-Impurity C	NMT 0.1%	0.00%	0.00%	0.00%	0.00%	0.00%	0.00%
-Impurity H	NMT 0.2%	0.00%	0.00%	0.00%	0.00%	0.00%	0.00%
-Impurity I	NMT 0.5%	0.1%	0.1%	0.1%	0.1%	0.1%	0.1%
-Unspecified impurities	NMT 0.25%	<0.1%	<0.1%	<0.1%	<0.1%	<0.1%	<0.1%
-Total impurities	NMT 1.5%	0.0%	0.0%	0.0%	<1.5%	<1.5%	<1.5%
Assay (HPLC)	90.0%—110.0%	100.4%	101.9%	100.1%	104.6%	103.9%	105.5%
Uniformity of dosage units	AV ≤ 15	6	13	10	13.2	9.6	8.8
Microbiology							
-TAMC	NMT 10^3^ CFU/g	<5 CFU/g	<5 CFU/g	<5 CFU/g	<5 CFU/g	<5 CFU/g	<5 CFU/g
-TYMC	NMT 10^2^ CFU/g	<5 CFU/g	<5 CFU/g	<5 CFU/g	<5 CFU/g	<5 CFU/g	<5 CFU/g
-E. coli	Absent	Absent	Absent	Absent	Absent	Absent	Absent
Dissolution	≥ 80% at 30 min						
−05 min		86.7%	91.8%	76%	74.4%	74.7%	79.9%
−10 min		93.3%	99.8%	98.8%	91.6%	88.5%	94.5%
−15 min		95.9%	101.1%	101.4%	96.1%	99.7%	101%
−20 min		96.4%	101.2%	98.8%	99.6%	102.1%	102.2%
−30 min		94.9%	101%	97.6%	99.3%	103.2%	103%
Disintegration	< 30 min	3 min	2 min	2 min	3 min	3 min	3 min

### 3.2 Stability studies

Final results, at the end of storage for 6 months on accelerated conditions and 12 months on long-term stability conditions, complied with the preset product specifications. All stability data are shown in Supplementary Tables S1–S4 (Supplementary Table S1: 35 mg capsules accelerated conditions, Supplementary Table S2: 250 mg capsules accelerated conditions, Supplementary Table S3: 35 mg capsules long-term conditions, Supplementary Table S4: 250 mg capsules long-term conditions). Only at the 9 months time-point of the long-term stability study, an out of specification result was found for two of the three 35 mg batches on assay content: 83.8%, 84.1%, and 93.1% (specification: 90%–110%). An investigation was performed but no root cause could be found. Subsequent results at 12 months showed that all results were within specification again. Supplementary Tables S1, S3 show that the assay content of the 35 mg capsules had a high variation, but no simultaneous changes are seen in degradation product. Supplementary Tables S2, S4 show that the assay content of the 250 mg capsules was overall relatively high with an average of approximately 104%, but still within the specification of 90%–110%.

**FIGURE 1:**
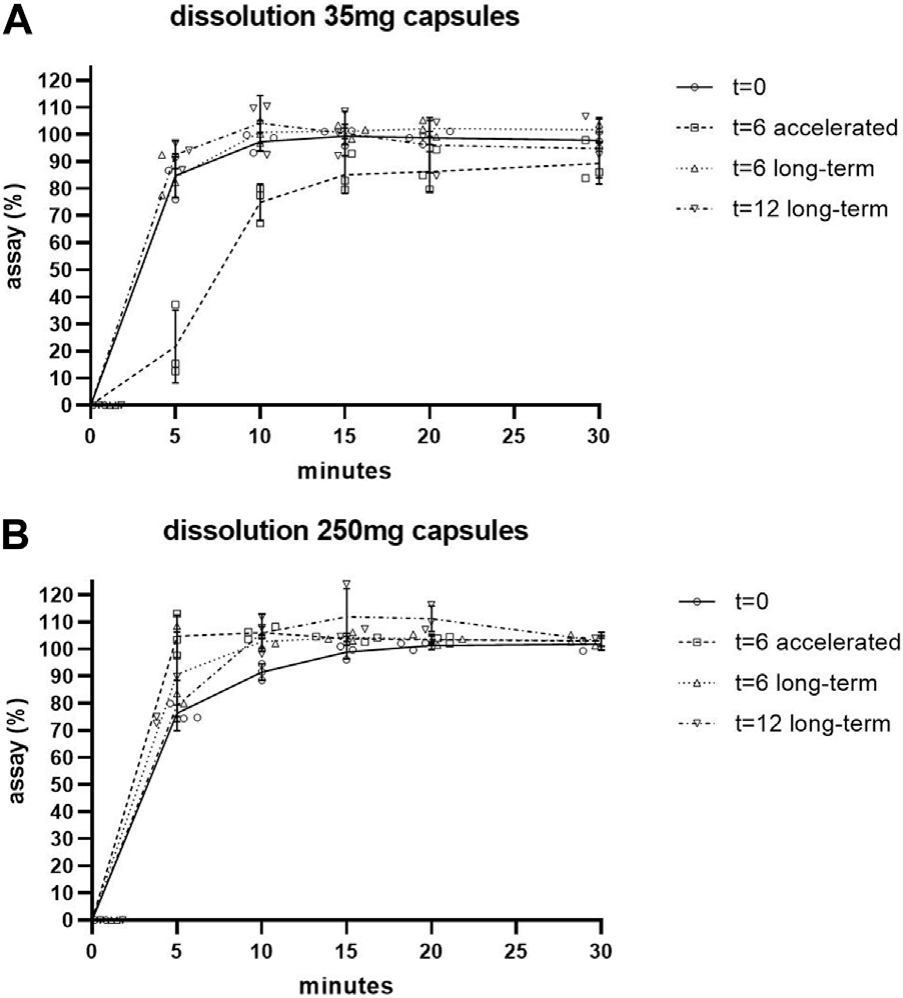
Dissolution of 35 mg **(A)** and 250 mg **(B)** capsules during stability studies. Individual values shown. SD shown as error bars.

The dissolution of the capsules remained fast during the stability program, the results are shown in Figure 1 and in Supplementary Tables S1–S4. The specification of ≥80% dissolution within 30 min was reached after 10 min for the 250 mg capsules, both long-term and accelerated conditions, and for the 35 mg capsules long-term conditions (Supplementary Tables S2–S4). For the 35 mg capsules accelerated condition this point was reached after 30 min for all three batches (Supplementary Tables S1). The inter-batch variation that was found in assay could also be seen in the assay percentages that are calculated in the dissolution test, both for the 35 mg and the 250 mg capsules, at all applicable time points in the stability studies.

Other results, as shown in Supplementary Tables S1–S4, showed that related substances had not increased and no unknown impurities had formed. No changes were detected in microbiological results. Uniformity of dosage units and disintegration also remained compliant throughout the stability program.

## 4 Discussion

From the results of the product validation we conclude that we have developed robust and high quality CDCA capsules in doses suitable for treatment of patients with CTX. The products comply to the set specifications and to national compounding guidelines and EU GMP guidelines. As expected, no impurities arise during the manufacturing process and during stability studies.

The results of the stability studies show that the 250 mg capsules are very stable and therefore a shelf life of 12 months is justified. The results of the 35 mg capsules are less uniform due to the 9 months assay results being below specification. No explanation for this outlier in results could be found. The analytical method has been validated with a high accuracy and an uneven distribution is ruled out as the in process controls show a consistently low RSD based on weight. Future ongoing stability studies are needed to determine if these out of specification results were incidental. Stability issues are not likely as no related substances were increased and no unknown peaks were detected. An analytical error is suspected (for instance in weighing, capsule emptying or sample processing), but could not be confirmed. As we were not able to find a plausible explanation, the shelf life of the 35 mg capsules and all other doses with exception of 250 mg, was set at 6 months.

Overall it can be concluded that the pharmacy compounded CDCA capsules are of high quality and stability. It is often thought that pharmacy preparations are of lower quality compared to commercially manufactured drugs. Using our pharmacy compounded CDCA capsules as an example, we demonstrated that pharmacy compounded drugs are a qualitative and affordable alternative and essential to assure treatment of patients in situations where a commercial drug is unavailable or inaccessible, without compromising on pharmaceutical quality. Furthermore they have an added value in customizing medication dosages to suit individual patients. The Amsterdam UMC pharmacy has supplied more than 60 patients with pharmacy compounded CDCA capsules and around 15% of the patients have received a capsule dose other than 250 mg. For these patients, the personalized compounding of alternative doses is an additional advantage.

This paper offers comprehensive guidance to other pharmacies, enabling them to create high-quality products in a straightforward manner. With this we hope to improve the availability of currently inaccessible drugs through the practice of pharmacy compounding, thereby facilitating improved patient care.

## Data Availability

The original contributions presented in the study are included in the article/Supplementary Material, further inquiries can be directed to the corresponding author.
