# Supplementary material for: Product development and quality of pharmacy compounded chenodeoxycholic acid capsules for Dutch cerebrotendinous xanthomatosis patients
Source: Front Pharmacol. 2023 Oct 17;14:1264997. doi: 10.3389/fphar.2023.1264997 (PMC10616945; doi:10.3389/fphar.2023.1264997)
Supplement: Supplementary file 1 [file DataSheet1.docx]

**Supplementary Table 1.** Stability data CDCA 35 mg capsules accelerated conditions (40°C ±2°C and 75%RH ±5%RH).

| **Test** | **Specification** | **T=0** | | | **T=3** | | | **T=6** | | |
| --- | --- | --- | --- | --- | --- | --- | --- | --- | --- | --- |
|  |  | Batch 1 | Batch 2 | Batch 3 | Batch 1 | Batch 2 | Batch 3 | Batch 1 | Batch 2 | Batch 3 |
| Appearance | Clear capsule with white to broken white powder | Complies | Complies | Complies | Complies | Complies | Complies | Complies | Complies | Complies |
| Identity (HPLC) | Positive | Positive | Positive | Positive | Positive | Positive | Positive | Positive | Positive | Positive |
| Related substances (HPLC)  -Impurity A  -Impurity B  -Impurity C  -Impurity H  -Impurity I  -Unspecified impurities  -Total impurities | Max. 1%  Max. 0.5%  Max. 0.1%  Max. 0.2%  Max. 0.5%  Max. 0.25%  Max. 1.5% | 0.00%  <0.05%  0.00%  0.00%  0.1%  <0.1%  0.0% | 0.00%  <0.05%  0.00%  0.00%  0.1%  <0.1%  0.0% | 0.00%  <0.05%  0.00%  0.00%  0.1%  <0.1%  0.0% | 0.00%  <0.05%  0.00%  0.00%  0.1%  <0.1%  <1.5% | 0.00%  <0.05%  0.00%  0.00%  0.1%  <0.1%  <1.5% | 0.00%  <0.05%  0.00%  0.00%  0.1%  <0.1%  <1.5% | 0.00%  <0.05%  0.00%  0.00%  0.1%  <0.1%  <1.5% | 0.00%  <0.05%  0.00%  0.00%  0.1%  <0.1%  <1.5% | 0.00%  <0.05%  0.00%  0.00%  0.1%  <0.1%  <1.5% |
| Assay (HPLC) | 90.0 – 110.0% | 100.4% | 101.9% | 100.1% | 95.2% | 100.1% | 97.1% | 92.2% | 100.5% | 99.4% |
| Uniformity of dosage units | AV ≤ 15 | 6 | 13 | 10 | - | - | - | 10 | 15 | 12 |
| Microbiology  -TAMC  -TYMC  -E. coli | Max 10^3^ CFU/g  Max 10^2^ CFU/g  Absent | <5 CFU/g  <5 CFU/g  Absent | <5 CFU/g  <5 CFU/g  Absent | <5 CFU/g  <5 CFU/g  Absent | <5 CFU/g  <5 CFU/g  Absent | <5 CFU/g  <5 CFU/g  Absent | <5 CFU/g  <5 CFU/g  Absent | <5 CFU/g  <5 CFU/g  Absent | <5 CFU/g  <5 CFU/g  Absent | <5 CFU/g  <5 CFU/g  Absent |
| Dissolution  -05 min  -10 min  -15 min  -20 min  -30 min | ≥ 80% at 30 min | 86.7%  93.3%  95.9%  96.4%  94.9% | 91.8%  99.8%  101.1%  101.2%  101.0% | 76.0%  98.8%  101.4%  98.8%  97.6% | - | - | - | 12.6%  80.0%  79.6%  79.7%  86.1% | 15.4%  77.6%  93.0%  94.4%  98.0% | 37.2%  67.2%  83.0%  84.9%  83.9% |
| Disintegration | < 30 min | 3 min | 2 min | 2 min | - | - | - | <30 min | <30 min | <30 min |

**Supplementary Table 2.** Stability data CDCA 250 mg capsules accelerated conditions (40°C ±2°C and 75%RH ±5%RH).

| **Test** | **Specification** | **T=0** | | | **T=3** | | | **T=6** | | |
| --- | --- | --- | --- | --- | --- | --- | --- | --- | --- | --- |
|  |  | Batch 1 | Batch 2 | Batch 3 | Batch 1 | Batch 2 | Batch 3 | Batch 1 | Batch 2 | Batch 3 |
| Appearance | Clear capsule with white to broken white powder | Complies | Complies | Complies | Complies | Complies | Complies | Complies | Complies | Complies |
| Identity (HPLC) | Positive | Positive | Positive | Positive | Positive | Positive | Positive | Positive | Positive | Positive |
| Related substances (HPLC)  -Impurity A  -Impurity B  -Impurity C  -Impurity H  -Impurity I  -Unspecified impurities  -Total impurities | Max. 1%  Max. 0.5%  Max. 0.1%  Max. 0.2%  Max. 0.5%  Max. 0.25%  Max. 1.5% | 0.00%  <0.05%  0.00%  0.00%  0.1%  <0.1%  0.0% | 0.00%  <0.05%  0.00%  0.00%  0.1%  <0.1%  0.0% | 0.00%  <0.05%  0.00%  0.00%  0.1%  <0.1%  0.0% | 0.00%  <0.05%  0.00%  0.00%  0.1%  <0.1%  <1.5% | 0.00%  <0.05%  0.00%  0.00%  0.1%  <0.1%  <1.5% | 0.00%  <0.05%  0.00%  0.00%  0.1%  <0.1%  <1.5% | 0.00%  <0.05%  0.00%  0.00%  0.1%  <0.1%  <1.5% | 0.00%  <0.05%  0.00%  0.00%  0.1%  <0.1%  <1.5% | 0.00%  <0.05%  0.00%  0.00%  0.1%  <0.1%  <1.5% |
| Assay (HPLC) | 90.0 – 110.0% | 104.6% | 103.9% | 105.5% | 104.5% | 106.3% | 104.8% | 102.9% | 101.9% | 105.5% |
| Uniformity of dosage units | AV ≤ 15 | 13.2 | 9.6 | 8.8 | - | - | - | 6.8 | 5.4 | 14.2 |
| Microbiology  -TAMC  -TYMC  -E. coli | Max 10^3^ CFU/g  Max 10^2^ CFU/g  Absent | <5 CFU/g  <5 CFU/g  Absent | <5 CFU/g  <5 CFU/g  Absent | <5 CFU/g  <5 CFU/g  Absent | <5 CFU/g  <5 CFU/g  Absent | <5 CFU/g  <5 CFU/g  Absent | <5 CFU/g  <5 CFU/g  Absent | <5 CFU/g  <5 CFU/g  Absent | <5 CFU/g  <5 CFU/g  Absent | <5 CFU/g  <5 CFU/g  Absent |
| Dissolution  -05 min  -10 min  -15 min  -20 min  -30 min | ≥ 80% at 30 min | 74.4%  91.6%  96.1%  99.6%  99.3% | 74.7%  88.5%  99.7%  102.1%  103.2% | 79.9%  94.5%  101.0%  102.2%  103.0% | - | - | - | 103.4%  108.3%  104.2%  104.0%  103.0% | 113.1%  106.3%  104.6%  104.5%  104.9% | 97.7%  103.6%  102.7%  102.0%  101.1% |
| Disintegration | < 30 min | 3 min | 3 min | 3 min | - | - | - | <30 min | <30 min | <30 min |

**Supplementary Table 3.** Stability data CDCA 35 mg capsules long-term conditions (25°C ±2°C and 60%RH ±5%RH).

| **Test** | **Specification** | **T=0** | | | **T=3** | | | **T=6** | | | **T=9** | | | **T=12** | | |
| --- | --- | --- | --- | --- | --- | --- | --- | --- | --- | --- | --- | --- | --- | --- | --- | --- |
|  |  | Batch 1 | Batch 2 | Batch 3 | Batch 1 | Batch 2 | Batch 3 | Batch 1 | Batch 2 | Batch 3 | Batch 1 | Batch 2 | Batch 3 | Batch 1 | Batch 2 | Batch 3 |
| Appearance | Clear capsule with white to broken white powder | Complies | Complies | Complies | Complies | Complies | Complies | Complies | Complies | Complies | Complies | Complies | Complies | Complies | Complies | Complies |
| Identity (HPLC) | Positive | Positive | Positive | Positive | Positive | Positive | Positive | Positive | Positive | Positive | Positive | Positive | Positive | Positive | Positive | Positive |
| Related substances (HPLC)  -Impurity A  -Impurity B  -Impurity C  -Impurity H  -Impurity I  -Unspecified impurities  -Total impurities | Max. 1%  Max. 0.5%  Max. 0.1%  Max. 0.2%  Max. 0.5%  Max. 0.25%  Max. 1.5% | 0.00%  <0.05%  0.00%  0.00%  0.1%  <0.1%  0.0% | 0.00%  <0.05%  0.00%  0.00%  0.1%  <0.1%  0.0% | 0.00%  <0.05%  0.00%  0.00%  0.1%  <0.1%  0.0% | 0.00%  <0.05%  0.00%  0.00%  0.1%  <0.1%  <1.5% | 0.00%  <0.05%  0.00%  0.00%  0.1%  <0.1%  <1.5% | 0.00%  <0.05%  0.00%  0.00%  0.1%  <0.1%  <1.5% | 0.00%  <0.05%  0.00%  0.00%  0.1%  <0.1%  <1.5% | 0.00%  <0.05%  0.00%  0.00%  0.1%  <0.1%  <1.5% | 0.00%  <0.05%  0.00%  0.00%  0.1%  <0.1%  <1.5% | <1.00%  <0.50%  <0.10%  <0.20%  0.1%  <0.1%  <1.5% | <1.00%  <0.50%  <0.10%  <0.20%  0.1%  <0.1%  <1.5% | <1.00%  <0.50%  <0.10%  <0.20%  0.1%  <0.1%  <1.5% | 0.00%  <0.50%  0.00%  0.00%  0.1%  <0.1%  <1.5% | 0.00%  <0.50%  0.00%  0.00%  0.1%  <0.1%  <1.5% | 0.00%  <0.50%  0.00%  0.00%  0.1%  <0.1%  <1.5% |
| Assay (HPLC) | 90.0 – 110.0% | 100.4% | 101.9% | 100.1% | 96.6% | 94.9% | 103.2% | 93.4% | 98.3% | 93.5% | 93.1% | 83.8% | 84.1% | 103.1% | 91.9% | 98.5% |
| Uniformity of dosage units | AV ≤ 15 | 6 | 13 | 10 | - | - | - | 9 | 13 | 7 | - | - | - | 9 | 9 | 14 |
| Microbiology  -TAMC  -TYMC  -E. coli | Max 10^3^ CFU/g  Max 10^2^ CFU/g  Absent | <5 CFU/g  <5 CFU/g  Absent | <5 CFU/g  <5 CFU/g  Absent | <5 CFU/g  <5 CFU/g  Absent | <5 CFU/g  <5 CFU/g  Absent | <5 CFU/g  <5 CFU/g  Absent | <5 CFU/g  <5 CFU/g  Absent | <5 CFU/g  <5 CFU/g  Absent | <5 CFU/g  <5 CFU/g  Absent | <5 CFU/g  <5 CFU/g  Absent | <10 CFU/g  <10 CFU/g  Absent | <10 CFU/g  <10 CFU/g  Absent | <10 CFU/g  <10 CFU/g  Absent | <10 CFU/g  <10 CFU/g  Absent | <10 CFU/g  <10 CFU/g  Absent | <10 CFU/g  <10 CFU/g  Absent |
| Dissolution  -05 min  -10 min  -15 min  -20 min  -30 min | ≥ 80% at 30 min | 86.7%  93.3%  95.9%  96.4%  94.9% | 91.8%  99.8%  101.1%  101.2%  101.0% | 76.0%  98.8%  101.4%  98.8%  97.6% | - | - | - | 92.6%  105.4%  101.7%  105.4%  104.9% | 77.7%  96.9%  98.4%  99.2%  96.7% | 82.3%  100.3%  103.5%  102.0%  103.8% | - | - | - | 86.9%  92.4%  92.1%  84.7%  85.4% | 96.8%  109.7%  100.7%  99.0%  92.4% | 94.1%  110.5%  108.6%  104.5%  106.7% |
| Disintegration | < 30 min | 3 min | 2 min | 2 min | - | - | - | <30 min | <30 min | <30 min | - | - | - | <30 min | <30 min | <30 min |

**Supplementary Table 4.** Stability data CDCA 250 mg capsules long-term conditions (25°C ±2°C and 60%RH ±5%RH).

| **Test** | **Specification** | **T=0** | | | **T=3** | | | **T=6** | | | **T=9** | | | **T=12** | | |
| --- | --- | --- | --- | --- | --- | --- | --- | --- | --- | --- | --- | --- | --- | --- | --- | --- |
|  |  | Batch 1 | Batch 2 | Batch 3 | Batch 1 | Batch 2 | Batch 3 | Batch 1 | Batch 2 | Batch 3 | Batch 1 | Batch 2 | Batch 3 | Batch 1 | Batch 2 | Batch 3 |
| Appearance | Clear capsule with white to broken white powder | Complies | Complies | Complies | Complies | Complies | Complies | Complies | Complies | Complies | Complies | Complies | Complies | Complies | Complies | Complies |
| Identity (HPLC) | Positive | Positive | Positive | Positive | Positive | Positive | Positive | Positive | Positive | Positive | Positive | Positive | Positive | Positive | Positive | Positive |
| Related substances (HPLC)  -Impurity A  -Impurity B  -Impurity C  -Impurity H  -Impurity I  -Unspecified impurities  -Total impurities | Max. 1%  Max. 0.5%  Max. 0.1%  Max. 0.2%  Max. 0.5%  Max. 0.25%  Max. 1.5% | 0.00%  <0.05%  0.00%  0.00%  0.1%  <0.1%  <1.5% | 0.00%  <0.05%  0.00%  0.00%  0.1%  <0.1%  <1.5% | 0.00%  <0.05%  0.00%  0.00%  0.1%  <0.1%  <1.5% | 0.00%  <0.05%  0.00%  0.00%  0.1%  <0.1%  <1.5% | 0.00%  <0.05%  0.00%  0.00%  0.1%  <0.1%  <1.5% | 0.00%  <0.05%  0.00%  0.00%  0.1%  <0.1%  <1.5% | 0.00%  <0.05%  0.00%  0.00%  0.1%  <0.1%  <1.5% | 0.00%  <0.05%  0.00%  0.00%  0.1%  <0.1%  <1.5% | 0.00%  <0.05%  0.00%  0.00%  0.1%  <0.1%  <1.5% | <1.00%  <0.50%  <0.10%  <0.20%  0.1%  <0.1%  <1.5% | <1.00%  <0.50%  <0.10%  <0.20%  0.1%  <0.1%  <1.5% | <1.00%  <0.50%  <0.10%  <0.20%  0.1%  <0.1%  <1.5% | 0.00%  <0.50%  0.00%  0.00%  0.1%  <0.1%  <1.5% | 0.00%  <0.50%  0.00%  0.00%  0.1%  <0.1%  <1.5% | 0.00%  <0.50%  0.00%  0.00%  0.1%  <0.1%  <1.5% |
| Assay (HPLC) | 90.0 – 110.0% | 104.6% | 103.9% | 105.5% | 103.9% | 104.9% | 103.8% | 104.5% | 102.4% | 104.5% | 103.5% | 103.6% | 104.0% | 104.1% | 103.8% | 103.8% |
| Uniformity of dosage units | AV ≤ 15 | 13.2 | 9.6 | 8.8 | - | - | - | 9.9 | 8.2 | 14.1 | - | - | - | 6.7 | 7.5 | 9.3 |
| Microbiology  -TAMC  -TYMC  -E. coli | Max 10^3^ CFU/g  Max 10^2^ CFU/g  Absent | <5 CFU/g  <5 CFU/g  Absent | <5 CFU/g  <5 CFU/g  Absent | <5 CFU/g  <5 CFU/g  Absent | <5 CFU/g  <5 CFU/g  Absent | <5 CFU/g  <5 CFU/g  Absent | <5 CFU/g  <5 CFU/g  Absent | <5 CFU/g  <5 CFU/g  Absent | <5 CFU/g  <5 CFU/g  Absent | <5 CFU/g  <5 CFU/g  Absent | <10 CFU/g  <10 CFU/g  Absent | <10 CFU/g  <10 CFU/g  Absent | <10 CFU/g  <10 CFU/g  Absent | <10 CFU/g  <10 CFU/g  Absent | <10 CFU/g  <10 CFU/g  Absent | <10 CFU/g  <10 CFU/g  Absent |
| Dissolution  -05 min  -10 min  -15 min  -20 min  -30 min | ≥ 80% at 30 min | 74.4%  91.6%  96.1%  99.6%  99.3% | 74.7%  88.5%  99.7%  102.1%  103.2% | 79.9%  94.5%  101.0%  102.2%  103.0% | - | - | - | 83.6%  102.1%  103.8%  103.7%  102.7% | 108.6%  105.4%  106.2%  105.5%  105.3% | 79.9%  100.8%  103.1%  101.5%  101.3% | - | - | - | 72.6%  107.9%  104.6%  110.1%  105.6% | 89.8%  98.2%  107.3%  107.1%  103.9% | 75.0%  111.8%  124.0%  116.3%  99.3% |
| Disintegration | < 30 min | 3 min | 3 min | 3 min | - | - | - | <30 min | <30 min | <30 min | - | - | - | <30 min | <30 min | <30 min |
